# China’s economic development quality grows faster than economic quantity

**DOI:** 10.1371/journal.pone.0289399

**Published:** 2023-07-28

**Authors:** Guangyue Xu, Haoyun Dong, Xiaojiang Shi

**Affiliations:** 1 Institute of Ecological Civilization Economy, School of Economics, Henan University, Kaifeng Henan, 475004, China; 2 School of Finance, Henan Finance University, Zhengzhou Henan, 450000, China; 3 School of College English Teaching and Research, Henan University, Kaifeng Henan, 475004, China; Loughborough University, UNITED KINGDOM

## Abstract

It is known that the rapid progress of the Chinese economy is perceived to be aligned more towards increasing the economic quantity (GDP) rather than its quality primarily owing to a large amount of carbon emission, increased environmental pollution, and high resource consumption. However, such perception comes with no single evidence or study that compares growth rates between the quantity and quality of China’s economic development. Such a comparative study could provide useful information to the Chinese government and concerned agencies to manage and promote sustainable economic development in China. Here we developed a comprehensive economic development quality evaluation indicator system to study China’s progress on economic quality from 1978 to 2017 and compare it with GDP growth rates over the same period. Results indicate that during the period 1978–2017, China’s economic development quality index has increased significantly, higher than the average annual growth rate of GDP during the same period. This higher growth rate of Chinese economic development quality could be attributed to the country’s achievements in tackling environmental pollution, narrowing the income gap, promoting technological progress and innovation, improving economic efficiency, maintaining social stability, and improving social welfare. We suggest the world re-evaluate China’s economic development achievement and growth patterns that can provide a reference for other developing countries.

## 1. Introduction

Since the economic reform in 1978, China, the largest developing country, has been globally recognized as one of the largest economies in the world. After four decades of rapid economic development since the reform and opening up in 1978, China’s GDP exceeded 90 trillion yuan in 2018, accounting for 15.9% of the world economy and contributing to almost 27.5% of the world’s current economic growth rate, 24.4% higher than in 1978 (National Bureau of Statistics, 2019). However, global challenges that are relevant to China, such as uneven resource distribution, environmental pollution, low economic efficiency, and structural imbalance have instigated the need for a paradigm shift in the focus of economic development from quantity to quality growth. As such, high-quality economic development has been a major focus of the international academic community since the end of the 20th century [1–3]. Every single country is promoting economic growth that fosters both economic well-being and improved quality of life of its population. Therefore, pursuing the improvement of economic development quality, a direct measure of people’s quality of life and social welfare, has become the main agenda of global sustainable development. High-quality economic development is an innovation-driven and multi-dimensional economic growth mode of common development. It is a growth model with technological innovation as the leading factor, high added value as the core, quality as the leading factor, making economic aggregate become effective economic aggregate, promoting industrial upgrading, and promoting comprehensive and sustainable development of economic construction, social construction, and ecological civilization construction. For a developing and emerging economy like China, high-quality economic development provides an important reference for measuring economic development and evaluating whether or not the country has completed the sustainable economic development process and if yes to which level the sustainable economic development has achieved.

Although China has made leap-forward progress in GDP, the substantial improvement in the quantity of economic growth has also brought with it some problems, such as excessive resource consumption, high environmental costs, poor stability, large differences in income distribution, insufficient improvement of people’s welfare, and imbalances in urban and rural development [4]. Considered itself to be at the late stage of industrialization [5], the Chinese economic development process has moved to a stage considered to be a “new normal” that focuses on changing from high to medium-high economic growth. As a result, the conventional quantity development model that only pursues fast-growing GDP in the past is being transformed into ones that foster continuous improvement of economic development quality. However, it is still commonly believed that over the past few decades China fared poorly in promoting economic development quality.

This paper will calculate the economic development quality development of China in the past few decades by constructing the economic development quality evaluation index system, so as to more intuitively study the quality level of China’s economic development in recent decades. At the national or regional level, the construction of economic quality index system and the application of economic development quality system have attracted extensive interest. Some scholars believe that the measurement of the quality of economic development should be multidimensional, including economic growth, political system, religious belief, legal order, people’s health and education level [1] or broader consideration of social development, ecological and environmental costs, improvement of economic efficiency, structural optimization, economic and social stability, welfare improvement and innovation level [6–9].

Some international scholars have also explored the impact of regional environment and the promotion of sustainable economic development from individual aspects such as trade openness, technological innovation, the use of renewable energy, green human resources, national policies, green innovation, etc. [10–16].

Meanwhile, since the COVID-19 pandemic, many scholars have explored the impact of epidemic diseases on social sustainable development. There is no doubt that the prevalence of COVID-19 has brought negative economic and emotional impacts to society and people. However, COVID-19 has promoted the reform of the public health sector due to the global public health crisis. In order to meet the market demand during the period when movements is restricted, the rapid progress of automation, mechanization and digital Industry 4.0 technology can help the company develop significantly. This technological progress and computer digitization provide people with a perspective that can improve environmental, economic and social sustainability [17,18].

Although the above studies fully express the connotation of economic development quality, they did not compare the temporal progress of China’s economic development quality with economic quantity. Moreover, the conventional economic development quality indicator system lacks indicators in scale development, which may lead to an incomplete and biased assessment of economic quality. A comprehensive assessment of economic development quality and its comparison with the quantity of economic growth could provide useful information to the Chinese government and concerned agencies to monitor and manage sustainable economic development in China.

Although the above studies fully express the connotation of economic development quality, they did not compare the temporal progress of China’s EDQ with economic quantity. Moreover, the conventional EDQ indicator system lacks indicators in scale development, which may lead to an incomplete and biased assessment of economic quality. A comprehensive assessment of EDQ and its comparison with the quantity of economic growth could provide useful information to the Chinese government and concerned agencies to monitor and manage sustainable economic development in China.

To address this important gap, for the first time, we employ a comprehensive economic development quality evaluation system to measure China’s economic development quality from 1978 to 2017 and compared its growth rate with that of GDP, which is commonly used as a measure for economic quantity. Our comprehensive economic development quality evaluation system for China advances previous economic development quality indicator systems by adding several previously ignored key indicators such as the economic scale. We then carried out a general evaluation, multi-dimensional analysis, and temporal analysis of China’s economic development quality from 1978 to 2017. To the best of our knowledge, this is the first-ever comparison between the growth rates of Chinese economic development quality and GDP.

We first redefined the main aspects of economic development quality to integrate the seven dimensions of sustainable economic development, namely, scale development (SCAD), stable development (STAD), efficiency development (EFD), structural development (STRD), innovative development (IND), green development (GRD), and people’s livelihood development (PLD). Under this framework, we constructed an economic development quality evaluation system with 36 characterization indicators to make it more measurable, comparable, and controllable, unlike the existing economic development quality indicator systems. We then studied the fluctuation and temporal patterns of China’s economic development quality index from 1978 to 2017 through the combination of principal component analysis and the entropy method. Second, to answer whether the rapid growth of the economic quantity of China was achieved at the expense of economic quality, we compared the growth rates of economic development quality and GDP of China from 1978 to 2017. Our results contradict the common perception that China’s economy is more quantitative centric, as we provide strong evidence that the historic economic development quality index growth rates in China have exceeded the GDP growth rates.

## 2.Methods

### 2.1 The reconstruction of China’s economic development quality evaluation indicators system

Based on the existing research on the quality index system of economic development, following the index system to evaluate China’s economic development quality constructed by Xu [19], we also re-constructed a more comprehensive and wide-ranging economic development quality evaluation indicator system from: the seven dimensions of scale development (SCAD), stable development (STAD), efficiency development (EFD), structural development (STRD), innovative development (IND), green development (GRD) and people’s livelihood development (PLD), including 36 concrete characterization indicators. Specifically, there are 2, 3, 5, 7, 4, 6, and 9 characterization indicators for SCAD, STAD, EFD, STRD, IND, GRD, and PLD, respectively. It is worth noting that the labor productivity, energy efficiency, and other indicators are calculated in Table 1, which lists all 7 dimensional indicators, 36 characterization indicators. Some calculation formulas of characterization indicators, indicator attributes, units, and data sources are detailed in Appendix A.1 in S1 Appendix.

**Table 1:** Evaluation indicator system of economic development quality.

Dimensions	Evaluating indicators	Number	Attribute	Data sources
SCAD	Economic aggregate	1	+	China Statistical Yearbook
	Economic growth	2	+	China Statistical Yearbook
STAD	Growth fluctuation	3	−	China Statistical Yearbook
	Price fluctuation	4	−	China Statistical Yearbook
	Unemploy- ment	5	−	China Statistical Yearbook
EFD	Labor efficiency	6	+	China Statistical Yearbook
	Investment efficiency	7	+	China Statistical Yearbook
		8	+	China Statistical Yearbook
	Cultivated land efficiency	9	+	China Statistical Yearbook
	Energy efficiency	10	+	China Statistical Yearbook
STRD	Industrial structure	11	+	China Statistical Yearbook
	Industrializa- tion level	12	+	China Statistical Yearbook
	Urbanization level	13	+	China Statistical Yearbook
	Financial development	14	+	China Statistical Yearbook
	Trade Development	15	+	China Statistical Yearbook
	Income structure	16	−	China Statistical Yearbook
	Energy structure	17	+	China Statistical Yearbook
IND	R&D	18	+	China Statistical Yearbook on Science and Technology
		19	+	China Statistical Yearbook on Science and Technology
	Invention &Creation	20	+	China Statistical Yearbook on Science and Technology
		21	+	China Statistical Yearbook on Science and Technology
GRD	Environmen- tal pollution	22	−	China Statistical Yearbook on Environment
		23	−	China Statistical Yearbook on Environment
		24	−	China Statistical Yearbook on Environment
	Pollution scale	25	−	China Statistical Yearbook on Environment
		26	−	China Statistical Yearbook on Environment
		27	−	China Statistical Yearbook on Environment
PLD	Quality of life	28	−	China Statistical Yearbook /Statistical Data Compilation of the 65th Anniversary of China
		29	+	China Statistical Yearbook
		30	+	China Statistical Yearbook
		31	−	China Statistical Yearbook
		32	−	China Statistical Yearbook
	People’s livelihood service guarantee	33	+	China Statistical Yearbook
		34	+	China Statistical Yearbook
		35	+	China Statistical Yearbook
		36	+	China Statistical Yearbook

Scale development (SCAD) includes two aspects: economic aggregate and economic growth. GDP is still an indispensable and important indicator to measure the quality of economic development. The high-quality development of the economy does not represent the stagnation and slow development of GDP. The perfect high-quality development is also consistent with the high-speed economic development. Therefore, we choose "GDP" and "GDP growth rate" to reflect the "quantity" and "speed" of economic growth respectively.

Stable development (STAD) includes economic stability and social stability. For the measurement of economic stability, we choose "economic growth volatility" and "price index volatility" as two indicators. At the same time, we reflect the degree of social stability through the "unemployment rate" of the society. A higher unemployment rate will also lead to higher social instability, and will also weaken economic development.

Efficiency development (EFD) is the core force to promote the economic development quality. As the main factor of production, the efficiency of labor, capital, land and energy use determines the level of social and economic output. Therefore, we choose "labor productivity", "loan productivity", "investment productivity", "cultivated land productivity" and "energy productivity " to measure the efficiency of economic development.

Structural development (STRD) means the coordination and optimization of economic and social structures. From a macro perspective, the adjustment, transformation and upgrading of the economic structure need to promote the optimization and improvement of the industrial structure. We use the ratio of the contribution rate of the tertiary industry’s output value to GDP and the contribution rate of the secondary industry’s output value to the GDP refers to the industrial structure. If the ratio rises gradually, the industrial structure will gradually improve. Meanwhile, we choose the index of “output value of non-agricultural industry/GDP” as the industrialization rate, the higher the ratio, the higher the level of industrialization of the country. The fruits of economic development should ultimately make the people benefit fairly. The reduction of urban-rural income gap is also an important manifestation of optimizing the urban-rural dual structure. Therefore, we use the "urban-rural income ratio" to reflect the distribution structure. A high-quality economy is bound to be an open economy. The higher the degree of opening to the outside world, the more conducive it is to promote industrial upgrading and high-quality development. Therefore, we use "total export-import volume /GDP" to examine the level of trade openness. The financial development level of a country represents the vitality of its economic development, we choose "added value of financial industry / GDP" to describe the financial development level. The optimization of the energy structure refers to the improvement of the utilization rate of clean energy. It is an important measure of energy modernization to make clean energy the main energy. Therefore, the proportion of non-fossil energy in the total energy consumption refers to the energy structure.

Science and technology are the fundamental driving force for promoting historical change and economic development. The purpose of innovative development (IND) is to examine the importance that the country and society attach to scientific and technological innovation and the value that scientific and technological innovation products and related economic activities bring to social and economic growth. Therefore, we measure the level of innovation and development in terms of scientific and technological investment and invention and creation. "R&D expenditure/GDP" and "full-time equivalent of R&D personnel" were selected to measure scientific and technological investment. "Patent application and authorization amount" and "technology contract turnover/GDP" were selected to measure inventions and creations. The more authorized patent applications represent the higher innovation enthusiasm and innovation level of the whole country. The higher proportion of technology contract turnover indicates that more innovative achievements are translated into practical technology applications, which helps to promote the upgrading of industrial structure and economic growth.

Green development (GRD) represents environmental sustainability in the process of economic development. Because industrial pollution is one of the main sources of pollutants in China, we choose "industrial wastewater discharge amount per unit of GDP ", "industrial waste gas discharge amount per unit of GDP " and "industrial solid waste production quantity per unit of GDP" to reveal the intensity of environmental pollution caused by economic growth. And in order to more intuitively observe the degree of environmental pollution caused by production activities, we have added the total emission indicators of these three industrial wastes.

People’s livelihood development (PLD) is to share the results of economic development with the people, reflect the "people-oriented" economic development idea, and is also the ultimate goal of social and national economic development, so that people can generally enjoy the benefits of economic growth. Therefore, we will examine the development of people’s livelihood from the aspects of people’s quality of life and people’s livelihood service guarantee.

### 2.2 Data collection and process

The data corresponding to the indicators is critical to understanding them. The indicator data of dimensions of SCAD, STAD, EFD, STRD, and PLD mainly came from China Statistical Yearbook (1979–2018) and Statistical Data Compilation of the 65^th^anniversary of the founding of new China, the indicator data of IND comes from China Statistical Yearbook on Science and Technology (1991–2018), and the indicator data of GRD came from China Statistical Yearbook on Environment (1998–2018).

For the indexes with price factors, such as tical Yearbook on Environment (1998–2018). d the d" economic development idea, and is also the ultimate goal of social and national er unit GDP”, “industrial waste gas discharge per unit GDP”, “industrial solid waste production per unit GDP”, “per capita GDP” and other index data, we took 1978 as the base period and used the CPI to reduce the GDP in the index calculation formula (GDP/CPI) to convert it into actual data.

However, there are still difficulties to be overcome in the process of data collection, such as the lack of data. For missing data in some years, we adopt linear interpolation method to estimate. Such as “investment productivity” (1978–1979), “R&D expenditure/GDP”(1978–1994), “full-time equivalent of R&D personnel”(1978–1990), “patent application and authorization amount”(1978–1979), “technology contract turnover/GDP” (1978–1990), “industrial waste gas discharge amount”(2016–2017), “Engel coefficient of urban residents”(1979), “social crime rate” (1978–1997), “education fund/GDP”(1978–1990), “urban endowment insurance coverage”(1978–1988) and “urban unemployment insurance coverage”(1978–1991). It’s important to note here that although the missing years of some indicators were more than 10 years, the weight of these indicators in the comprehensive index is small, and the focus of the calculation results was to describe the changing trend of the overall economic development quality, the estimation of these missing data would not cause evident deviation to the calculation of the results. Such indicators include “R & D expenditure/GDP” in 1978–1994, “R & D personnel full-time equivalent” in 1978–1990, “national technology contract turnover / GDP” in 1978–1990, “crime rate” in 1978–1997, “education fund/GDP” in 1978–1990, “urban basic endowment insurance coverage” in 1978–1988 and “urban unemployment insurance coverage” in 1978–1991.

### 2.3 Principal component analysis method and the entropy method

Generally speaking, for the objective data, we should focus on the objective evaluation driven by data to avoid subjective interference. The principal component analysis method reduces dimension and simplifies data as the starting point, transforms multiple indicators with original correlation into a few comprehensive indicators with better representativeness, solves the problem of information overlap between the original indicators, and creates new indicators to retain the information of the original data to the maximum extent, which is usually expressed as the linear combination of the original indicators. The entropy method is also an objective method for index weighting, whose principle is to calculate the information entropy of the indicator and determine the weight of the indicator according to the influence of the relative change degree of the indicator on the whole system such that the indicator with large relative change degree has large weight [20]. We combined the principal component analysis method and the entropy method to determine the indicator weight. We used SPSS 21.0 to extract the main components of each dimension in China’s economic development quality evaluation system, simplify the evaluation system, and eliminate the correlation between the indicators. Appendix A.2 in S1 Appendix shows the statistical characteristics of each dimension index. Under the requirement of the cumulative contribution rate reaching at least 85%, the SCAD dimension only contains two indicators and the STAD dimension only contains three indicators, so principal component analysis was no longer carried out for these two dimensions. And all indicators of the dimensions are directly used as principal components to give weight by entropy method, and the cumulative contribution rate is 100%. Based on this, the entropy method is used to give objective weight (see Appendices A.2, A.3, A.4 in S1 Appendix) to the obtained principal component, which makes the weight result more objective and scientific. It is worth noting that for reverse indicators such as “economic growth volatility”, “price index volatility”, “urban registered unemployment rate”, “urban-rural income ratio”, “industrial wastewater discharge amount per unit of GDP”, “industrial waste gas discharge amount per unit of GDP”, “industrial solid waste production quantity per unit of GDP”, “industrial wastewater discharge amount”, “industrial waste gas discharge amount”, “industrial solid waste production quantity”, “Engel coefficient of urban residents population mortality” and “social crime rate”, the reciprocal was taken to transform them into a positive indicator before principal component analysis.

### 2.4 Empirical mode decomposition

Empirical mode decomposition is a data processing method proposed by Huang, et al. [21] and its basic idea is to decompose irregular wave data into several single wave components and one trend component. The wave components of different scales are defined as Intrinsic Mode Function (IMF), that is, prototype wave = ∑*IMFs* + residual (see Eq (1)).


$$X(t)=\sum_{i=1}^{n}IMFi(t)+r(t)$$ (1)

The IMF component decomposed from the original signal can be linear and nonlinear, and the IMF component must meet two conditions: 1) the number of extreme points and zero points of the IMF component are the same or no more than a difference of one and 2) the upper and lower envelope lines are locally symmetric about the zero axes [21]. Using Matlab R2018a software, we conducted an empirical mode decomposition analysis on China’s economic development quality index, through which two IMF components (IMF1 and IMF2) and one trend were obtained.

## 3.Results

### 3.1 The growth of China’s economic development quality: 1978–2017

**Fig 1:**
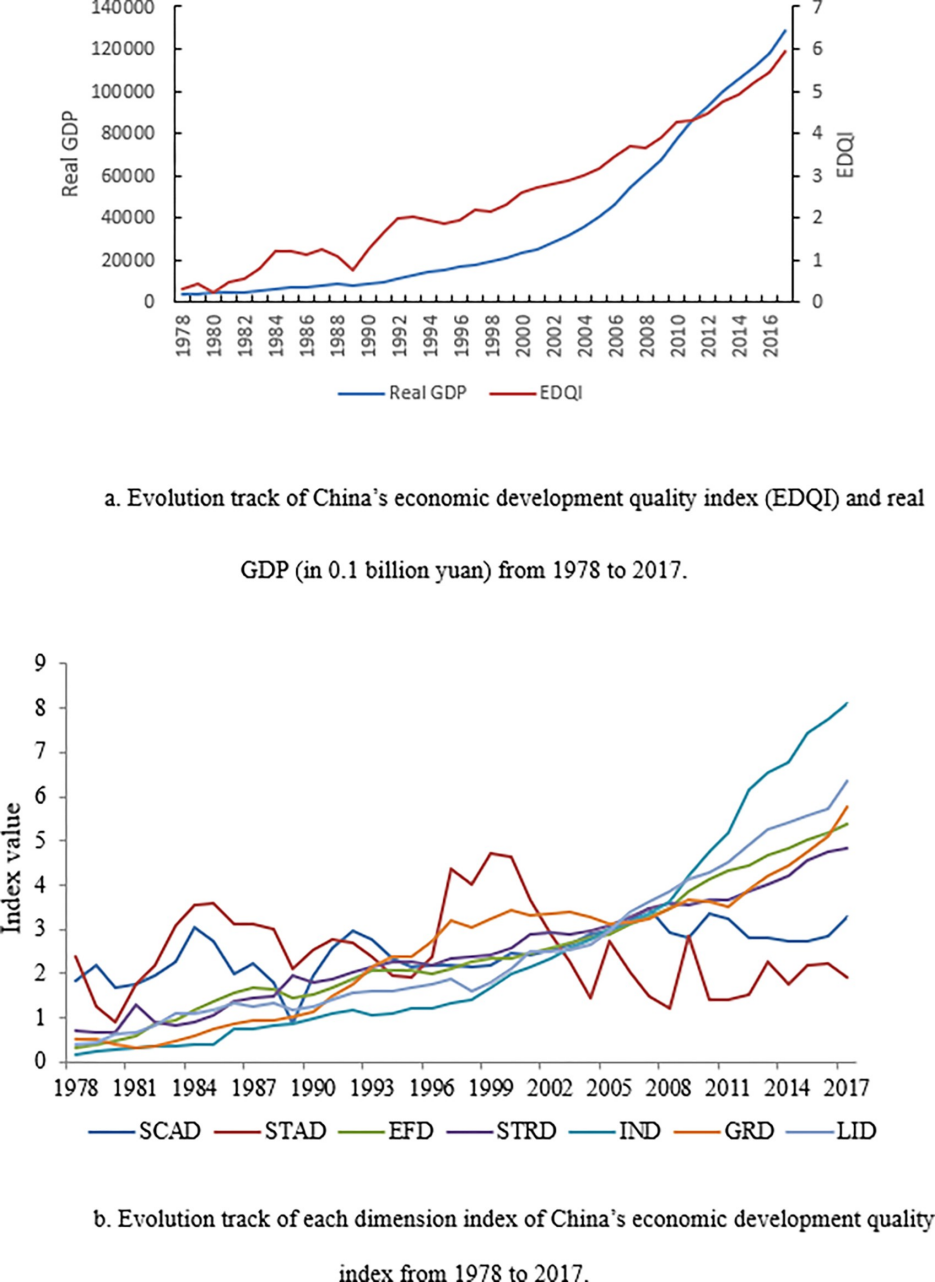
a. Evolution track of China’s economic development quality index (EDQI) and real GDP (in 0.1 billion yuan) from 1978 to 2017. b. Evolution track of each dimension index of China’s economic development quality index from 1978 to 2017.

The results indicate that China’s economic development quality has greatly improved since the 1970s, as indicated by the upward trends of the economic development quality index and most of its dimensions (Fig 1A and 1B). Specifically, economic development quality index increased from 0.30 in 1978 to 5.95 in 2017 (i.e. an increase of about 18.82 times) with an average annual growth rate of 10.33%, which is greater than the average annual growth rate (9.63%) of China’s real GDP from 1978 to 2017 (see Fig 1A). Nonetheless, the growth rate of economic development quality is not monotonic, as indicated by slight year to year fluctuations, which could be attributed to the wide variations in annual growth rates of its dimensions (Fig 1A and 1B).

In terms of dimensionality, the scale development index (SCADI) showed very high volatility, especially before 1995, but then began to rise steadily until 2006 when it started to decline with a decline in GDP growth, as a result of the economic crisis. Similar to SCADI, the stable development index (STADI) showed large fluctuations with an upward trend from 1980 to 1985 to a downward trend until 1995, after which it began to rise. The innovative development index (INDI) showed the largest increase of 0.16 in 1978 to 8.09 in 2017 (i.e. increased by 50 times), with an average annual growth rate of 10.61% and an average annual increase of about 0.20. The efficiency development index (EFDI) showed a steady rise and increased from 0.31 in 1978 to 5.37 in 2017, with an increase of 16.29 times and an average annual growth rate of about 7.98%. The people’s livelihood development index (PLDI) had a stable growth rate and increased by 14.38 times from 1978 (0.41) to 2017 (6.3), with an average annual growth rate of 7.66%, indicating that the quality of people’s livelihood development has been relatively stable and distinctly improved. Meanwhile, the green development index (GRDI) also increased by 10 times from 1978 to 2017, with an annual growth rate of 6.95%. The structural development index (STRDI) increased from 0.73 in 1978 to 4.85 in 2017 with an increase of 5.66 times and an average annual growth rate of 6.07%, this is due in large part to the clean transformation of the energy structure.

Moreover, China’s GDP has been in an upward trend during most of the 1978–2017 period, and in most of the years, it was positive except for negative growth in 1989. Starting in 1992, its growth rate showed a steady trend until 2006. In 2007, the growth rate of GDP showed a relatively downward trend, and it was always lower than 10% after 2011, suggesting the economic growth was slowing down gradually. The average growth rate of GDP was about 9.63% from 1978 to 2017, which was slightly lower than China’s economic development quality index growth rate during the same period. In addition, China’s economic development quality index is gradually rising unlike GDP growth rates that this slightly slowing down in recent years.

Because of the differences in the construction of indicator system and measurement methods, the evaluation results of economic development quality obtained by us and the existing literature will also be different. We choose the paper calculation results of Chao and Ren [6], Shi and Li [22] to compare with the results of this paper, and analyze the reasons for the differences.

Chao and Ren [6] measured the China’s economic development quality from 1978 to 2007 by establishing an evaluation system that includes the four dimensions of economic growth, such as structure, stability, welfare and achievement distribution, resource utilization and ecological environment. The final result is that the economic development quality index increased from -3.0374 in 1978 to 3.6072 in 2007, with an average annual growth rate of 0.02%. The index basically shows an upward trend in volatility, which is roughly consistent with our judgment on the evolution trend of economic development quality. The following is a discussion of the difference in the index direction of a specific year.

Shi and Li [22] built an evaluation system that includes five dimensions: innovation-driven, coordinated development, green ecology, openness and stability, and sharing and harmony to measure China’s economic development quality in 2000–2017. The results show that China’s economic development quality has shown a steady upward trend since 2000, with economic development quality index below—1 in 2000 and rising to close to 2 in 2017. The growth range of the index is large from 2012 to 2016, with an average annual growth of 0.271. However, the growth rate slowed down significantly in 2017, with an average annual growth of only 0.05. However, the calculation result of this paper is that the average annual growth of economic development quality index is 0.25 in 2012–2016, while the average annual growth in 2017 is 0.51, with a significant increase. There is a big difference between this paper and Stan and Li Peng (2019) in the growth rate in 2017. The reason is that the evaluation system constructed in this paper has added the dimension of SCAD more than the evaluation system constructed by Stan and Li Peng (2019). The SCAD has declined to a certain extent in 2013–2015, and then increased significantly in 2017, resulting in the increase of the economic development index in 2017.

### 3.2 The fluctuation of economic development quality in China

**Fig 2:**
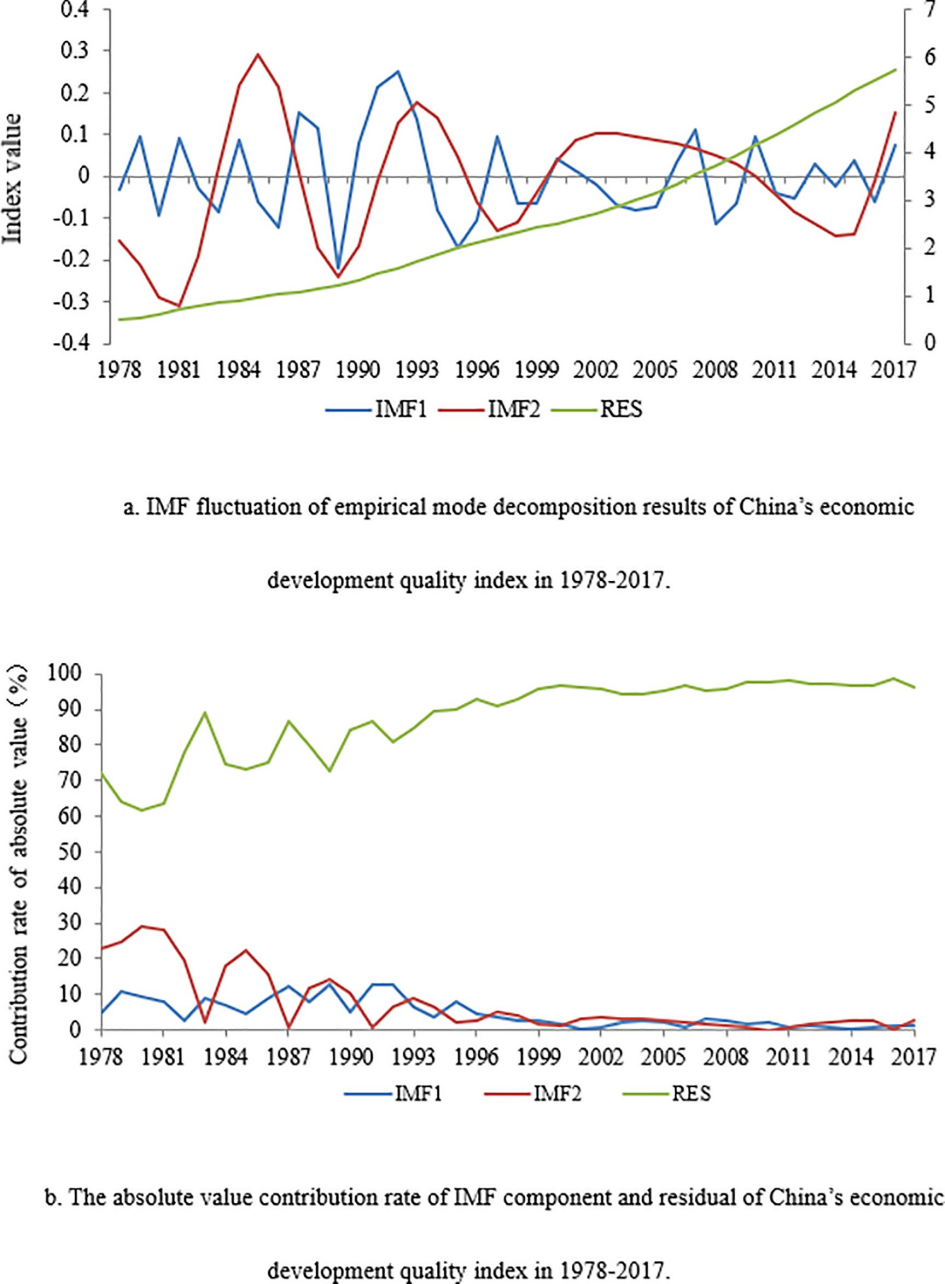
a. IMF fluctuation of empirical mode decomposition results of China’s economic development quality index in 1978–2017. b. The absolute value contribution rate of IMF component and residual of China’s economic development quality index in 1978–2017.

China’s economic development quality index followed a cyclical pattern from 1978 to 2017, as indicated by Fig 2, which shows the absolute value contribution rate of the Intrinsic Mode Function (IMF) component and the residual of China’s economic development quality index in 1978–2017. Fig 2A shows the two IMF components (that is IMF1 and IMF2) obtained by decomposing economic development quality index with the Empirical mode decomposition method. The fluctuation periods of the two IMF components are different, representing different time scales.

IMF1 shows a short period fluctuation of 2–6 years. Specifically, China’s economic development quality has roughly passed 11 short cycles since 1978, with an average of 3.45 years per cycle, including three 2-year cycles, four 3-year cycles, two 4-year cycles, and two 6-year cycles. Before 1986, there were two 2-year cycles and one 3-year cycle, and the amplitude was relatively small; from 1986 to 1995, there were one 3-year cycle and one 6-year cycle, and the amplitude was relatively large; from 1995 to 2016, the amplitude decreased, and there was one 3-year cycle from 1995 to 1998, one 6-year cycle from 1998 to 2004, and two 4-year cycles from 2004 to 2012. Finally, there were two 2-year cycles between 2012 and 2016, and the amplitude of the latter two cycles was further reduced compared with the previous period, and the cycle lasting time was also shortened. Based on this, we can say that after 2012, the cycle lasting time has shortened, the volatility of economic development quality index has reduced, and the impact of the short cycle on economic development quality has decreased, which has made the trend of economic development quality gradually stable in recent years. The reason for IMF1 showing such a high fluctuation is due to the high volatility of SCAD and STD dimensions. After 2004, SCAD began to fluctuate with a small amplitude, with a 4-year (2005–2009) and 3-year (2009–2012) fluctuations, and the declining amplitude during this period resulted in a relatively stable short period rule of economic development quality indexI. Since 2012, SCAD has not fluctuated much and STAD only experienced two short-period fluctuations with a small amplitude, which is consistent with the fluctuation law of IMF1.

IMF2 follows a long period of fluctuation. Its periodic fluctuation shows a gradually weakening pattern with increased lasting time and gradually declining amplitude and influence of a long period on economic development quality. Specifically, China’s economic development quality experienced at least three long cycles from 1978 to 2017, with an average of 11 years in each cycle consisting of two 8-year cycles (1981–1989 and 1989–1997) and a 17-year cycle (1997–2014). During these cycles, peak IMF2 values were reached in 1985 (0.2926), 1993 (0.1777), and 2002 (0.1035) with four minimum values in the years 1981 (-0.3116), 1989 (-0.2407), 1997 (-0.1285), and 2014 (-0.1418), indicating a recent decline in the frequency and amplitude of economic development quality in China. During 2014–2017, a new long-period peak of economic development quality started with a peak value of 0.1510 in 2017, which is higher than the peak value of the previous cycle, indicating that the new long-period might have a more distinct impact on China’s economic development quality. The reason for the first cycle of economic development quality index is potentially due to the peak values of SCAD in 1984 and STAD in 1985, which began to decline to new low values in 1989, which resulted in the first cycle of economic development quality index. Similarly, their periodic fluctuation during 1989–1995 led to the second cycle of IMF2. Since 1997, the amplitude of IMF2 was reduced to a great extent with longer cycle time indicating the long-term fluctuation of China’s economic development quality gradually slowed down after 1997. This is because in 1997, influenced by the Asian financial crisis and the domestic deflation trend, China’s economic growth rate declined, and the economic stability plummeted. Therefore, the Chinese government issued a series of relevant policies to expand domestic demand, deepened reform and opening up, speeded up economic restructuring, This resulted in a gradual rise in the demand for consumption and investment to strengthen the mechanism of independent economic growth and a steady rise in the economic scale and economic growth, which reduced the volatility of SCAD and then became more and more stable from 1999. After 2012, the volatility of SCAD and STAD both decreased, and the growth rate of the other five dimensions gradually accelerated, especially IND, which made the third cycle move to a higher peak (i.e. 2014).

Fig 2B shows the absolute value contribution rate of IMF component and residual of China’s economic development quality index from 1978 to 2017. The significantly larger value of the absolute contribution rate of residual value (over 60%) compared to the two IMF components suggest that the trend component has been the leading factor of economic development quality improvement and the steady increase trend is more influential than the periodic fluctuation trend, which indicates that China has been in a period of improving the economic development quality. At the beginning of the reform and opening up, China’s economic development quality showed great volatility, largely because the economic development was in the exploratory stage, the economic system was extremely imperfect, and a large number of reform projects and economic construction projects were abandoned. At the same time, there were also a large number of poor farmers, job-seeking or unemployed graduates, and returned educated youth, which affected the stability of economic and social development. With the deepening of reform and opening up, the fluctuation of power quality gradually decreased. After 1995, the proportion of the two economic development quality index’s volatility components was less than 10%, while the proportion of the trend component remained above 90%, which showed that the rising trend of China’s economic development quality was more and more obvious. Between the two volatility components, during 1978–1990, the long-term absolute value contribution rate was relatively large and a dominating factor, while in 1990–2000, the short-term absolute value contribution rate was dominant. After 2000, the trend of the absolute value contribution rate of the two volatility components showed no pattern (or flat), and the proportion of the two volatility components reduced to 0%. The cycle effect was further reduced, and thus China’s economic development quality entered a relatively stable growth period after or around year 2000.

## 4. Main conclusion and policy recommendations

### 4.1 Main conclusion

Using a novel economic development quality evaluation system, we assessed the multi-dimensional and comprehensive quantitative index of China’s economic development quality from 1978 to 2017 and then analyzed the temporal characteristics of economic development quality and present the first-ever comparison of the growth rates of quality and quantity of economic development and show that the average growth rate of Chinese economic development quality is higher than that of GDP. This breaks the traditional understanding of China’s economic development model that economic development in the past has resulted in high quantity growth and low quality of economic growth. However, according to conventional wisdom, China’s economic development model has always been centered on “economic quantity” or the “rapid growth of economic quantity”. The reason for this new understanding (or misunderstanding) is that the prior economic quality system was not comprehensive, as it excluded several important factors on the scale of the economy which were associated with the quality of economic growth.

What’s more, by comparing the evolution track of each dimension index and economic development quality index, all but SCAD and STAD showed an obvious increasing trend. IND showed the largest growth rates (increased by 50.07 times in 2018), indicating that China’s innovation level and innovation ability have been greatly improved. All seven dimensions have a positive driving effect on China’s economic development quality, of which STAD is the most volatile component though it contributes least among all to the improvement of economic development quality. Furthermore, China’s economic development quality which fluctuated greatly before 1995, has been on a more stable growth trend in recent years. After 1995, the proportion of fluctuation component accounts for less than 10% of the overall index trend, and economic development quality began to grow steadily. Starting in 2012, China’s economic development quality entered a new round of long-term cycles, and the impact of long-term cycles on economic development quality is expected to increase in the future.

Furthermore, through the empirical mode decomposition analysis of economic development quality index, it was found that China’s economic development quality with the previously highly fluctuation nature has more and more steady growth trend in recent year. The larger fluctuation period is mainly concentrated in the early stage of reform and opening up. However, with the continuous advancement of the reform and opening up process, the overall volatility of economic development quality has gradually decreased. After 1995, the proportion of fluctuation component accounts for less than 10% of the overall index trend, and economic development quality began to grow steadily. Starting in 2012, China’s economic development quality entered a new round of long-term cycles, and the impact of long-term cycles on economic development quality is expected to increase in the future.

Although the economic development quality and the scale of GDP development cannot be compared in absolute terms, the economic development quality grows faster than the quantity of economic development. This result is reflected in economics and can be explained as the economic development quality is a broader connotation. With the needs of the national economic transformation, the driving force of economic growth has undergone structural changes, from the original simple factor-driven and investment-driven to innovation-driven and environment-friendly coordinated development in many aspects. The original economic system and economic growth model cannot meet the new development requirements and provide continuous growth impetus for the new development stage, so the economic growth rate has slowed down. The development of other dimensions of the economic development evaluation system is to adapt to the needs of transformation, which is bound to improve rapidly. This situation will last for a period of time. In the later stage, with the continuous transformation, the quantity of economic growth will gradually establish a benign interaction mechanism with the quality of economic development, and the economic growth will appear a new form of high-quality and stable growth.

This paper explores the relationship between the quantity and quality of economic development, clarifies the connotation of the quality of economic development, confirms the achievements of China’s economic development quality progress, and clarifies the meaning of the fluctuation of the economic development quality index, providing reference significance for the high-quality development of China’s economy in the future.

### 4.2 Policy recommendations

Our findings put forward the two three suggestions for the improvement of China’s economic development quality.

First, we should make it clear on the key points to promote economic development quality in an all-around way, including scale, stability, efficiency, structure, innovation, green, and people’s livelihood development. Specifically, for GRD, EFD, and STRD with a large weight, we should continue to maintain their leading role in economic development quality. To improve GRD, China should further control the scale of industrial pollutant emissions, especially industrial waste gas emissions and industrial solid waste production, and significantly reduce its emission. To improve EFD, China should focus on improving the efficiency of capital utilization, optimizing the investment structure, improving the effectiveness and accuracy of investment, and enhancing loan productivity and investment productivity. To improve STRD, China should focus on the problem of the income gap between urban and rural areas, balancing urban and rural income in the process of promoting the development of urbanization, especially increasing the income level of rural residents and urban ordinary workers, expanding the scale of the middle class, and promoting the transformation from traditional agriculture to modern technology agriculture through the power of science and technology. With the development of a new normal mode and the transformation of the economy to high-quality development, the economy no longer only pursues high development speed. Hence SCAD should maintain a constant and stable speed at present, and the growth rate will remain slow in the appropriate developmental stage. To improve STAD, China should focus on controlling the unemployment and inflation rates, balancing the relationship between inflation and unemployment, and making the employment rate decrease to an ideal state, to alleviate the instability in economic and social development. To improve economic development quality by improving other dimensions, such as PLD, China needs to strengthen medical, educational, and health conditions, improve the legal system, and reduce population mortality and crime rate.

Second, China should accelerate the transformation of the extensive economic development mode and take the road of enhanced intensive development and implement the concept of intensive development in all aspects of economic development. Then China should accelerate the enhancement of economic agglomeration, population concentration, allocation optimization, resource conservation, and emission reduction. At the same time, China needs to increase its efforts to support national innovation to promote the coordinated development of multiple dimensions of economic development quality and improve the welfare level of economic development. By benefiting more and more people for the achievements of economic development, the state can promote more equitable distribution in the social field. Finally, sustainable economic and social development are key to improve China’s economic development quality in the long run.

Thirdly, Continuing to improve the quality of China’s development is the theme of China’s current and future economic and social development. China should take concrete action to gradually solve the problem of unbalanced and inadequate development in the economic and social fields, and clear up all kinds of obstacles to improving the quality of China’s economic development. Therefore, China must have the strategic resolve to promote the continuous improvement of the quality of economic development, not to be swayed by local and temporary disturbances. Improving the quality of economic development should be a basic concept for China’s medium and long term development and integrated into the country’s 14^th^ five-year plan and even 2050 national development plans, and thus ensure that China’s economic and social development track can continue to advance along the direction of high-quality development. Its aim is to meet the needs of the Chinese people for a better life, on the one hand, to achieve a high degree of harmony between man and nature, on the other hand, to achieve a high degree of harmony between man and man. On this basis, it will be more conducive for China and other countries to work together to build a community of human destiny and a community of life on earth. In this way, China will influence other countries, especially the developing countries, to embark on the road of overall improvement in the quality of economic development, thus promoting the process of improvement in the quality of economic development worldwide.

In a word, the quality model of China’s economic development has been proved by historical achievements. China will continue to maintain the state of quality growth driven by various forces in the quality of economic development, this will not only enable China to address its own problems of unbalanced and inadequate development in order to help achieve the great goal of rejuvenation of the Chinese nation, but will also help propel other countries onto the path of improving the quality of their economic development. This means that the path of improving the quality of China’s economic development is bound to continue to benefit the whole world.

## Supporting information

S1 Appendix (DOCX)
