# Supplementary material for: China’s economic development quality grows faster than economic quantity
Source: PLoS One. 2023 Jul 28;18(7):e0289399. doi: 10.1371/journal.pone.0289399 (PMC10381078; doi:10.1371/journal.pone.0289399)
Supplement: S1 Appendix — (DOCX) [file pone.0289399.s001.docx]

Appendix：

Appendix A.1 Evaluation indicator system of economic development quality (Detailed version)

| **Dimensions** | **Evaluating indicators** | **Num-ber** | **Characterization indicators and calculation formula** | **Unit** | **Attri-**  **Bute** | **Data sources** |
| --- | --- | --- | --- | --- | --- | --- |
| **SCAD** | Economic aggregate | 1 | GDP | Million yuan | + | China Statistical Yearbook |
|  | Economic growth | 2 | GDP growth =(Current year’s GDP - Last year’s GDP) / Last year's GDP × 100% | % | ＋ | China Statistical Yearbook |
| **STAD** | Growth fluctuation | 3 | Economic growth volatility =(Current year’s GDP growth - Last year’s GDP growth) / Last year’s GDP growth × 100% | % | － | China Statistical Yearbook |
|  | Price fluctuation | 4 | Price index volatility =(Current year’s CPI - Last year’s CPI)/ Last year’s CPI× 100% | % | － | China Statistical Yearbook |
|  | Unemploy-  ment | 5 | Urban registered unemployment rate | % | － | China Statistical Yearbook |
| **EFD** | Labor efficiency | 6 | Labor productivity = GDP / Number of workers | Ten thousand yuan / person | ＋ | China Statistical Yearbook |
|  | Investment efficiency | 7 | Loan productivity = GDP / Amount of loans used by financial institutions | - | ＋ | China Statistical Yearbook |
|  |  | 8 | Investment productivity=GDP / Total fixed asset investment of the whole society | - | ＋ | China Statistical Yearbook |
|  | Cultivated land efficiency | 9 | Cultivated land productivity =Gross value of agricultural output /Cultivated area | Million/1000 hectares | ＋ | China Statistical Yearbook |
|  | Energy efficiency | 10 | Energy productivity =GDP/Total energy consumption | Ten thousand yuan /One Ton of standard coal | ＋ | China Statistical Yearbook |
| **STRD** | Industrial structure | 11 | Contribution rate of tertiary industry to GDP/Contribution rate of secondary industry to GDP | - | ＋ | China Statistical Yearbook |
|  | Industrializa-  tion level | 12 | Output value of non-agricultural industry /GDP | % | ＋ | China Statistical Yearbook |
|  | Urbanization level | 13 | Urbanization rate = Urban population / Total Population | % | ＋ | China Statistical Yearbook |
|  | Financial development | 14 | Added value of financial industry / GDP | % | ＋ | China Statistical Yearbook |
|  | Trade Development | 15 | Total export-import volume /GDP | % | ＋ | China Statistical Yearbook |
|  | Income structure | 16 | Urban-rural income ratio =Urban per capita disposable income /Rural per capita disposable income | - | － | China Statistical Yearbook |
|  | Energy structure | 17 | Non-fossil energy consumption/Total energy consumption | % | ＋ | China Statistical Yearbook |
| **IND** | R&D | 18 | R&D expenditure /GDP | % | ＋ | China Statistical Yearbook on Science and Technology |
|  |  | 19 | Full-time equivalent of R&D personnel | Ten thousand people | ＋ | China Statistical Yearbook on Science and Technology |
|  | Invention &Creation | 20 | Patent application and authorization amount | - | ＋ | China Statistical Yearbook on Science and Technology |
|  |  | 21 | Technology contract turnover / GDP | % | ＋ | China Statistical Yearbook on Science and Technology |
| **GRD** | Environmen-  tal pollution | 22 | Industrial wastewater discharge amount per unit of GDP = Industrial wastewater dischargeamount /GDP | Standard cubic meter / yuan | － | China Statistical Yeabook on Environment |
|  |  | 23 | Industrial waste gas discharge amount per unit of GDP = Industrial waste gas discharge amount /GDP | Tons / Ten thousand yuan | － | China Statistical Yeabook on Environment |
|  |  | 24 | Industrial solid waste production quantity per unit of GDP = Industrial solid waste productionquantity /GDP | Tons / Ten thousand yuan | － | China Statistical Yeabook on Environment |
|  | Pollution scale | 25 | Industrial wastewater discharge amount | Hundred billion standard cubic meters | － | China Statistical Yeabook on Environment |
|  |  | 26 | Industrial waste gas discharge amount | Million tons | － | China Statistical Yeabook on Environment |
|  |  | 27 | Industrial solid waste productionquantity | Million tons | － | China Statistical Yeabook on Environment |
| **PLD** | Quality of life | 28 | Engel coefficient of urban residents | % | － | China Statistical Yearbook /Statistical Data Compilation of the 65th Anniversary of China |
|  |  | 29 | Engel coefficient of rural residents | Million yuan | + | China Statistical Yearbook |
|  |  | 30 | Per capita GDP | % | ＋ | China Statistical Yearbook |
|  |  | 31 | Population mortality | % | － | China Statistical Yearbook |
|  |  | 32 | Social crime rate =the people’s Procuratorate approves and decides to arrest the number of suspects /Total Population | % | － | China Statistical Yearbook |
|  | People's livelihood service guarantee | 33 | Education funds / GDP | % | ＋ | China Statistical Yearbook |
|  |  | 34 | Urban endowment insurance coverage =Number of people participating in endowment insurance /Urban population | Ten thousand yuan / person | ＋ | China Statistical Yearbook |
|  |  | 35 | Urban unemployment insurance coverage =Number of people participating in unemployment insurance /Urban population | - | ＋ | China Statistical Yearbook |
|  |  | 36 | Number of beds per 10000 medical institutions | - | ＋ | China Statistical Yearbook |

Appendix A.2 Statistical characteristics of dimensions

| Dimensions | Components | Eigen value | % of variance | Cumulative % of variance |
| --- | --- | --- | --- | --- |
| SCAD | 1 | —— | —— | 100 |
|  | 2 |  |  |  |
| STAD | 1 | —— | —— | 100 |
|  | 2 |  |  |  |
|  | 3 |  |  |  |
| EFD | 1 | 4.503 | 90.053 | 90.053 |
|  | 2 | 0.387 | 7.749 | 97.802 |
| STRD | 1 | 4.705 | 67.218 | 67.218 |
|  | 2 | 1.239 | 17.702 | 84.920 |
|  | 3 | 0.628 | 8.977 | 93.897 |
| IND | 1 | 3.821 | 95.522 | 95.522 |
|  | 2 | 0.132 | 2.292 | 98.814 |
| GRD | 1 | 4.732 | 78.868 | 78.868 |
|  | 2 | 0.787 | 13.115 | 91.983 |
| LID | 1 | 6.997 | 77.739 | 77.739 |
|  | 2 | 1.083 | 12.037 | 89.776 |
| EDQ | 1 | 5.336 | 76.229 | 76.229 |
|  | 2 | 0.976 | 13.937 | 90.165 |

Appendix A.3 Principal component coefficient and component weight of indicators

| Dimensions | Indicators | First principal component coefficient | First principal component weight | Second principal component coefficient | Second principal component weight | Weight |
| --- | --- | --- | --- | --- | --- | --- |
| SCAD | GDP | — | — | — | — | 0.4345 |
|  | GDP growth | — |  | — |  | 0.5654 |
| STAD | Economic growth volatility | — | — | — | — | 0.2769 |
|  | Priceindex volatility | — |  | — |  | 0.3294 |
|  | Urban registered unemployment rate | — |  | — |  | 0.3936 |
| EFD | Labor productivity | 0.449 | 0.9277 | 0.473 | 0.0723 | 0.4508 |
|  | Loan productivity | -0.411 |  | 0.764 |  | -0.3260 |
|  | Investment productivity | -0.454 |  | 0.254 |  | -0.4026 |
|  | Cultivated land productivity | 0.457 |  | 0.333 |  | 0.4477 |
|  | Energy productivity | 0.464 |  | 0.140 |  | 0.4403 |
| IND | R&D expenditure /GDP | 0.493 | 0.9634 | 0.710 | 0.0366 | 0.5009 |
|  | Full-time equivalent of R&D personnel | 0.507 |  | -0.221 |  | 0.4804 |
|  | Patent application and authorization amount | 0.497 |  | -0.644 |  | 0.4552 |
|  | Technology contract turnover / GDP | 0.503 |  | 0.168 |  | 0.4907 |
| GRD | Industrial wastewater discharge amount per unit of GDP | 0.399 | 0.8795 | -0.150 | 0.1205 | 0.3328 |
|  | Industrial waste gas discharge amount per unit of GDP | 0.421 |  | 0.203 |  | 0.3947 |
|  | Industrial solid waste production quantity per unit of GDP | 0.450 |  | -0.020 |  | 0.3934 |
|  | Industrial wastewater discharge amount | 0.283 |  | 0.869 |  | 0.3536 |
|  | Industrial waste gas discharge amount | -0.440 |  | 0.285 |  | -0.3526 |
|  | Industrial solid waste productionquantity | -0.433 |  | 0.317 |  | -0.3426 |
| LID | Engel coefficient of urban residents | 0.364 | 0.8586 | -0.113 | 0.1414 | 0.2965 |
|  | Engel coefficient of rural residents | 0.366 |  | 0.136 |  | 0.3332 |
|  | Per capita GDP | 0.370 |  | 0.150 |  | 0.3393 |
|  | Population mortality | -0.281 |  | -0.381 |  | -0.2953 |
|  | Social crime rate | -0.310 |  | 0.203 |  | -0.2378 |
|  | Education funds / GDP | 0.363 |  | -0.205 |  | 0.2830 |
|  | Urban endowment insurance coverage | 0.361 |  | -0.014 |  | 0.3076 |
|  | Urban unemployment insurance coverage | -0.212 |  | 0.753 |  | -0.0752 |
|  | Number of beds per 10000 medical institutions | 0.338 |  | 0.388 |  | 0.3448 |

Appendix A.4 Principal component coefficient and weight of indicators of STRD

| Dimensions | Indicators | First principal component coefficient | First principal component weight | Second principal component coefficient | Second principal component weight | Third principal component coefficient | Third principal component weight | Weight |
| --- | --- | --- | --- | --- | --- | --- | --- | --- |
| STRD | Contribution rate of tertiary industry to GDP/Contribution rate of secondary industry to GDP | 0.168 | 0.7468 | 0.703 | 0.1482 | -0.616 | 0.1050 | 0.1653 |
|  | Output value of non-agricultural industry /GDP | 0.452 |  | -0.135 |  | -0.015 |  | 0.3158 |
|  | Urbanization rate = Urban population / Total Population | 0.450 |  | 0.095 |  | 0.054 |  | 0.3558 |
|  | Added value of financial industry / GDP | 0.381 |  | 0.131 |  | 0.593 |  | 0.3664 |
|  | Total export-import volume /GDP | 0.363 |  | -0.404 |  | -0.303 |  | 0.1792 |
|  | Urban-rural income ratio | -0.356 |  | 0.412 |  | 0.347 |  | -0.1682 |
|  | Non-fossil energy consumption/Total energy consumption | 0.402 |  | 0.356 |  | 0.232 |  | 0.3773 |

Appendix A.5 Principal component coefficient and component weight of dimensions

| Dimensions | First principal component coefficient | First principal component weight | Second principal component coefficient | Second principal component weight | Weight |
| --- | --- | --- | --- | --- | --- |
| SCAD | 0.314 | 0.8552 | 0.023 | 0.1448 | 0.2718 |
| STAD | -0.122 |  | 0.969 |  | 0.0362 |
| EFD | 0.428 |  | 0.058 |  | 0.3745 |
| STRD | 0.423 |  | 0.066 |  | 0.3716 |
| IND | 0.420 |  | -0.050 |  | 0.3519 |
| GRD | 0.406 |  | 0.223 |  | 0.3799 |
| LID | 0.428 |  | -0.027 |  | 0.3618 |
